# Construction Notes

**Published:** 1895-08-31

**Authors:** 


### CONSTRUCTION NOTES.

### West of Scotland Convalescent Home.

The Seaside Home at Dunoon has been recently enlarged by the addition of a new wing, which will add 50 beds to the 200 the home previously contained. The cost of the new building, with furnishing, &c., has been nearly £5,000. At the inaugural ceremony Dr. Robert Perry stated that nearly 60,000 convalescents had been admitted during the 27 years that the hospital has existed. The formal opening of the new wing has taken place.

### New Infirmary at Paisley.

This new infirmary will be an imposing and handsome edifice, and as complete and modern in its internal arrangement and equipment as is possible. The site is in a commanding position in Calside. The infirmary will be situated in the centre of the grounds facing north, and having the principal entrance in the centre of the front of the building. The ground plan shows the infirmary partaking of the form of the letter E, running almost east and west. The long part of the building will contain only the corridors, and will have, projecting from it on the north or front side three blocks, that in the centre being the administrative block, a square handsome construction, in which is the main entrance. The block to the east side of this is a finely rounded building, containing circular wards, and that to the west a smaller building than either of the other two, containing nurses' rooms on the ground floor and doctors' bed-rooms above. The infirmary generally will consist of two storeys and attics, the eastern section of the building, as also the corridors, having basement flats. In the east wing of the buildings we find on the basement the stores, the porter's apartments, vapour bath, &c., on the south side of the corridor, while on the north side of this wing is a circular ward for twelve beds, duty-room for nurses, single ward, day-room, bath-room and other accommodation. On the ground floor is a ward containing sixteen beds for female medical cases, duty-room, single ward, store and recreation-rooms, with verandah, a circular ward for twelve beds, lavatories, &c. On the first floor is provided a ward for sixteen beds for female surgical cases, single-ward, duty-room, recreation-room, and verandah. On the north side of this same corridor another circular ward affords accommodation for twelve beds for female surgical cases. The Second Pavilion: The central arm of the E has on the ground floor a ward containing sixteen beds for male medical cases, and on the first floor a similar ward for male surgical cases, and on both floors are provided single wards, duty-rooms, recreation-rooms and verandahs, bath-rooms, lavatories, &c. In the Western Pavilion will be an accommodation of a similar kind, the large ward on the ground floor being—according to the plans—for male medical cases, and that above for male surgical cases. Between the Central and the Western Pavilions is a small structure projecting from the corridors and containing on the ground floor the doctors' and nurses' dining-rooms, on the first floor the chapel and chaplain's-room. The spaces between the three pavilions are to be preserved as recreation ground for the patients. In the administrative block to the front we find on the ground floor the vestibule and hall porter's office, visitors'-room, and doctors'-room; whilst leading from here are stairs to the first floor, where are situated the board-room, committee-room, doctors' sitting-room, &c. Forming part of this block is a structure containing on the basement floor casualty ward, &c.; on the ground floor, matron's apartments; on the first floor, operating-room, anæsthetic-room, doctors'-room for testing, &c.; entrance to all these apartments being from the corridors. The attics, which are confined to the principal line of buildings, contain at the east end servants' bed-rooms, and in the centre kitchen and servants' dining-hall. Throughout, the lighting is excellent and the heating and ventilating arrangements are on the most approved methods. The washhouses and laundry buildings are outside the main building. On the south side is placed the mortuary. The nurses' home is designed for the north-west corner of the grounds. These buildings, it is estimated, will cost £60,000 or £70,000. The plans have been prepared by Mr. T. Graham Abercrombie.

